# Datasets for genome assembly of six underutilized Indonesian fruits

**DOI:** 10.1016/j.dib.2018.12.070

**Published:** 2018-12-28

**Authors:** Deden Derajat Matra, Arya Widura Ritonga, Azis Natawijaya, Roedhy Poerwanto, Sobir, Ulfah Juniarti Siregar, Winarso Drajad Widodo, Eiichi Inoue

**Affiliations:** a Department of Agronomy and Horticulture, Faculty of Agriculture, Bogor Agricultural University, Indonesia; b Research and Development Division, Mekarsari Fruit Garden, Indonesia; c Department of Silviculture, Faculty of Forestry, Bogor Agricultural University, Indonesia; d College of Agriculture, Ibaraki University, Japan

## Abstract

Indonesia has a high genetic diversity of tropical fruits. However, studies on genomics are still very limited. In this data article, six underutilized Indonesian fruits were analyzed for the estimated genome size and partial data of genome assembly including *Artocarpus nangkadak* (*Artocarpus heterophyllus x Artocarpus integer)*, *Salacca sumatrana*, *Flacourtia inermis*, *Lansium domesticum*, *Pometia pinnata*, and *Syzygium samarangense*. These genome data may be used to construct molecular markers for plant systematics and breeding program of these species. Our genome data were sequenced paired-end libraries using BGISeq-500 and generated approximately 5 Gb of bases per species. The raw sequences have been deposited in the DNA Data Bank of Japan (DDBJ) under the DDBJ BioProject umbrella with accession number PRJDB7265 and to the DDBJ Read Archive for each species following *Artocarpus nangkadak* (DRA007398), *Salacca sumatrana* (DRA007394), *Flacourtia inermis* (DRA007395), *Lansium domesticum* (DRA007393), *Pometia pinnata* (DRA007396), *Syzygium samarangense* (DRA007397).

**Specifications table**

**Table:** 

Subject area	Agricultural and Biological Sciences
More specific subject area	Horticulture
Type of data	Whole Genome Sequencing (WGS) Data
How data were acquired	BGISeq-500 Sequencer
Data format	Raw Sequencing reads
Experimental factors	Young leaves were collected on liquid nitrogen and genomic DNA were extracted using DNeasy Plant Mini Kit
Experimental features	Genome sequencing was performed following BGISeq-500 protocol for WGS
Data source location	Cileungsi, Bogor, West Java, Indonesia (6°24′50.1′′S 106°59′05.7′′E)
Data accessibility	The raw sequences have been deposited in the DNA Data Bank of Japan (DDBJ) under the DDBJ BioProject umbrella with accession number PRJDB7265, and to the DDBJ Read Archive for each species following *Artocarpus nangkadak*DRA007398, *Salacca sumatrana*DRA007394, *Flacourtia inermis*DRA007395, *Lansium domesticum*DRA007393, *Pometia pinnata*DRA007396, *Syzygium samarangense*DRA007397
Related research article	T.K. Lim, Edible Medicinal and Non Medicinal Plants, vol. 3, Fruits. Springer, Netherlands, 2012.

**Value of the data**

• These data provide genomic data of six Indonesian underutilized fruits for genetic studies and breeding program.
• These data will be useful to obtain molecular markers such as microsatellite and single nucleotide polymorphisms for breeding and selection of new cultivars from six underutilized Indonesian fruits.
• These data will further be valuable for more complex studies on plant systematics among their species and genus.

## 1 Data

Many edible tropical fruits are native to South East Asia such as Indonesia, Malaysia, Philippines, and Thailand. Some underutilized fruits in Indonesia are important genetic resources for crop improvement, biomass, and food security [1]. In this data article, we analyzed genome size estimation and the draft genome assembly of six Indonesian underutilized fruits following *Artocarpus nangkadak* (*Artocarpus heterophyllus x Artocarpus integer)*, *Salacca sumatrana*, *Flacourtia inermis*, *Lansium domesticum*, *Pometia pinnata*, and *Syzygium samarangense*. The estimated genome size was analyzed using flow cytometry [2]. The genomes of the six Indonesian fruits were sequenced using paired-end libraries of BGISeq-500.

## 2 Experimental design, materials, and methods

### 2.1 Genome size estimation

The 1 cm^2^ of leaves was mixed with nuclei extraction buffer of CyStain UV Precise P (Cytotechs, Kandatsu, Japan). The nuclei were isolated from leaves using chopping method with razor blade and stained with staining buffer of CyStain UV Precise P (Cytotechs, Kandatsu, Japan). The stained nuclei were counted using Cyflow (Sysmex Partec, Gorlitz, Germany). The data were analyzed using FlowMax Software. The *Raphanus sativus* was used as plant reference for 2C DNA value estimation [2].

### 2.2 DNA extraction, whole genome sequencing and assembly

Genomic DNA was extracted from the young leaves using DNeasy Plant Mini Kit (Qiagen) following the protocol. The quality and quantity of DNA were checked by P360 Nanophotometer (Implen, München, Germany). Library quality was assessed on the Agilent Bioanalyzer 2100 system. The libraries were sequenced on the BGISeq-500 platform based on sequencing by synthesis with 100 bp paired-end reads (BGI, HongKong). The extracted genomic DNA was subjected to preparation of a paired-end library for genome sequencing using the BGISeq-500. After sequencing, the raw reads were filtered. Data filtering include removing adaptor sequences, contamination and low-quality reads from raw reads (Table 1).

**Table 1:** Estimated genome size, number of reads and DDBJ DRA Accession numbers of six underutilized Indonesian fruits.

No.	Species	2C DNA of pg (Mbp)	Number of reads	DDBJ DRA accession
1	*Artocarpus nangkadak*	0.53 (516.51)	58,861,118	DRA007398
2	*Salacca sumatrana*	1.37 (1336.34)	58,944,302	DRA007394
3	*Flacourtia inermis*	0.54 (524.50)	59,043,492	DRA007395
4	*Lansium domesticum*	0.64 (622.66)	61,010,166	DRA007393
5	*Pometia pinnata*	0.48 (468.63)	59,150,252	DRA007396
6	*Syzygium samarangense*	0.56 (548.33)	58,710,898	DRA007397

The assembly of reads from each species was performed through DDBJ Read Annotation Pipeline [3], [4] using ABySS 1.3.2 [5], Platanus 1.2.2 [6], SOAPdenovo 2.04-r240 [7], and Velvet 1.2.10 [8] with default parameters and the contigs have filtering minimum of 200 bp. The contig statistics from each assembler were calculated using Assembly-stat program [9] (Table 2). The contigs generated from the four assemblers will be made available at http://rujakbase.id.

**Table 2:** Number of contigs and N50 value from assembly of six underutilized Indonesian fruits.

No.	Species	ABySS		Platanus		SOAPdenovo		Velvet	
		NC	N50	NC	N50	NC	N50	NC	N50
1	*Artocarpus nangkadak*	252,762	228	52	6748	426,898	286	255,526	262
2	*Salacca sumatrana*	430,123	289	342	2098	579,111	522	659,362	501
3	*Flacourtia inermis*	923,769	380	117	1271	257,502	351	694,452	650
4	*Lansium domesticum*	908,488	385	110	18,612	421,289	327	455,010	654
5	*Pometia pinnata*	327,392	1560	131,211	2461	294,044	499	176,002	4241
6	*Syzygium samarangense*	663,163	423	175	8165	259,098	383	521,352	672

NC: number of contig.

### 2.3 Data accessibility

The raw read data were submitted to the DDBJ Read Archive (Table 1).
